# Supplementary material for: Tight bounds for the median of a gamma distribution
Source: PLoS One. 2023 Sep 8;18(9):e0288601. doi: 10.1371/journal.pone.0288601 (PMC10490949; doi:10.1371/journal.pone.0288601)
Supplement: S1 Appendix — (PDF) [file pone.0288601.s001.pdf]

Appendix

To approximate the standard gamma distribution CDF integral via partial summation of the series expansion

$$\frac{1}{\Gamma(k_i)} \int_0^{\nu_i} x^{k_i-1} e^{-x} dx = \frac{1}{\Gamma(k_i)} \sum_{n=0}^{\infty} \frac{(-1)^n \nu_i^{n+k_i}}{n!(n+k_i)} ,$$

we start with $n \leftarrow 0$, $r \leftarrow 1/\Gamma(k_i)$, and first term and initial sum $s \leftarrow t \leftarrow r\nu_i^{k_i}/k_i$; then iterate for $n \leftarrow n+1$, with magnitude ratio $r = \nu_i(n-1+k_i)/((n+k_i)n)$, new term $t \leftarrow -rt$, and sum $s \leftarrow s+t$. As explained in the Theorem L7 proof, when $r < 0.5$ and $n > 2\nu_i$ we take the error bound to be $e \leftarrow |t|$. When $s+e < 0.5$, terminate with CDF estimate s and error bound e, verifying the point bound $\nu_i < \nu(k_i)$.

These arrays of values follow the specified algorithm for the eight lower bounds used in Theorem L7; they terminate with success when the lower right-hand corner of the array is less than 0.5:

$i = 1 \quad k_i = 0.40 \quad \nu_i = 0.145 \quad \Gamma(k_i) = 2.21815$

n	r	t	s	e	$s+e$
0	0.450826	0.520591	0.520591	–	–
1	0.041429	−0.021567	0.499023	0.021567	0.520591
2	0.042292	0.000912	0.499935	0.000912	0.500847
3	0.034118	−0.000031	0.499904	0.000031	0.499935

$i = 2 \quad k_i = 0.44 \quad \nu_i = 0.177 \quad \Gamma(k_i) = 2.01319$

n	r	t	s	e	$s+e$
0	0.496724	0.526952	0.526952	−	−
1	0.054083	−0.028499	0.498452	0.028499	0.526952
2	0.052230	0.001489	0.499941	0.001489	0.501429
3	0.041849	−0.000062	0.499879	0.000062	0.499941

$i = 3 \quad k_i = 0.50 \quad \nu_i = 0.227 \quad \Gamma(k_i) = 1.77245$

n	r	t	s	e	$s+e$
0	0.564191	0.537612	0.537612	−	−
1	0.075667	−0.040679	0.496933	0.040679	0.537612
2	0.068100	0.002770	0.499703	0.002770	0.502473
3	0.054048	−0.000150	0.499553	0.000150	0.499703

$i = 4 \quad k_i = 0.60 \quad \nu_i = 0.315 \quad \Gamma(k_i) = 1.48919$

n	r	t	s	e	$s+e$
0	0.671506	0.559609	0.559609	−	−
1	0.118125	−0.066104	0.493506	0.066104	0.559609
2	0.096923	0.006407	0.499912	0.006407	0.506319
3	0.075833	−0.000486	0.499427	0.000486	0.499912

$i = 5 \quad k_i = 0.75 \quad \nu_i = 0.454 \quad \Gamma(k_i) = 1.22541$

n	r	t	s	e	$s+e$
0	0.816053	0.601796	0.601796	−	−
1	0.194571	−0.117092	0.484704	0.117092	0.601796
2	0.144455	0.016915	0.501618	0.016915	0.518533
3	0.110978	−0.001877	0.499741	0.001877	0.501618
4	0.089605	0.000168	0.499909	0.000168	0.500077
5	0.075009	−0.000013	0.499897	0.000013	0.499909

$i = 6 \quad k_i = 1.00 \quad \nu_i = 0.693 \quad \Gamma(k_i) = 1.00000$

n	r	t	s	e	$s+e$
0	1.000000	0.693000	0.693000	−	−
1	0.346500	−0.240124	0.452875	−	−
2	0.231000	0.055469	0.508344	0.055469	0.563813
3	0.173250	−0.009610	0.498734	0.009610	0.508344
4	0.138600	0.001332	0.500066	0.001332	0.501398
5	0.115500	−0.000154	0.499912	0.000154	0.500066
6	0.099000	0.000015	0.499928	0.000015	0.499943

$i = 7 \quad k_i = 1.50 \quad \nu_i = 1.182 \quad \Gamma(k_i) = 0.88622$

n	r	t	s	e	$s+e$
0	1.128388	0.966704	0.966704	−	−
1	0.709200	−0.685586	0.281117	−	−
2	0.422143	0.289415	0.570533	−	−
3	0.306444	−0.088690	0.481843	0.088690	0.570533
4	0.241773	0.021443	0.503286	0.021443	0.524729
5	0.200031	−0.004289	0.498997	0.004289	0.503286
6	0.170733	0.000732	0.499729	0.000732	0.500461
7	0.148992	−0.000109	0.499620	0.000109	0.499729

$i = 8 \quad k_i = 3.50 \quad \nu_i = 3.172 \quad \Gamma(k_i) = 3.32335$

n	r	t	s	e	$s+e$
0	0.300901	4.886771	4.886771	–	–
1	2.467111	−12.056207	−7.169436	–	–
2	1.297636	15.644573	8.475137	–	–
3	0.894667	−13.996678	−5.521541	–	–
4	0.687267	9.619450	4.097909	–	–
5	0.559765	−5.384629	−1.286720	–	–
6	0.473018	2.547024	1.260304	–	–
7	0.409986	−1.044245	0.216059	–	–
8	0.362022	0.378039	0.594098	0.378039	0.972138
9	0.324249	−0.122579	0.471520	0.122579	0.594098
10	0.293704	0.036002	0.507521	0.036002	0.543523
11	0.268476	−0.009666	0.497856	0.009666	0.507521
12	0.247280	0.002390	0.500246	0.002390	0.502636
13	0.229212	−0.000548	0.499698	0.000548	0.500246
14	0.213624	0.000117	0.499815	0.000117	0.499932
