# Supplementary material for: Tight bounds for the median of a gamma distribution
Source: PLoS One. 2023 Sep 8;18(9):e0288601. doi: 10.1371/journal.pone.0288601 (PMC10490949; doi:10.1371/journal.pone.0288601)
Supplement: Attachment — Submitted filename: Report - PONE- - D - 22 - 34782.pdf [file pone.0288601.s002.pdf]

Review Report

Tight bounds for the median of a gamma distribution

by

Richard F. Lyon

1 Summary

The paper deals with upper and lower bounds for the median of the standard gamma distribution. From a mathematical point of view, this is an interesting problem, because the median of a standard gamma distribution, as a function of its shape parameter k, has no known representation in terms of elementary functions. Many prior publications have considered this problem (Wilson and Hilferty, 1931; Chen and Rubin, 1986; Choi, 1994; Berg and Pedersen, 2006; Berg and Pedersen, 2008; Gaunt and Merkle, 2021; Lyon, 2021), but the bounds found by the author are tighter at low k than previously known bounds, and are proved valid over all $k > 0$. Some of the results proved in the article were conjectured in a previous work of the author (Lyon, 2021) published in Plos One. Thus, the paper aims at proved such conjectures and complementing the studies on the median of a standard gamma distribution.

The proves of paper results relying on previously known and new bounds that are tighter in some regions of the domain, and also relying on numerically transparent evaluations of the cumulative distribution function at a few points, using a convergent series. The strategy adopted by the author is to show that over various subsets of the k domain, with their union covering $(0, +\infty)$, there are other bounds that the author proves are between the new closed-form bounds and the true median. In this way, along the paper, the author derives several other new upper and lower bounds that are tighter over portions of the k domains.

The paper is completed with several graphs obtained by simulation on which the previous and new results are compared. Those graphs seem to me to be particularly valuable for fully understanding the article.

2 Recommendation

I appreciated reading this paper which presents clearly new bounds for the median of a standard gamma distribution. The paper is well written and clear. The problem addressed by the article is interesting and the results seem to be relevant to probability theory and could be potentially useful. However, the paper needs minor revisions before it can be accepted for publication. Thus, should the author answer adequately to my comments and suggestions below, then I would recommend the paper for publication in Plos One.

3 Comments and corrections

Below, typos, minor comments, or corrections are collected.

1. p.1-9: γ should be defined.
2. p.1-17: might be "...probability density function (PDF)..." instead of "...PDF...".
3. p.1-19: might be $x > 0$ instead of $x \geq 0$. Note that if $k = 1$ and $x = 0$, we have $p_1(0) = 0^0$, which is an indeterminate form.
4. p.1-23: might be "... cumulative distribution function (CDF)..." instead of "...CDF... ".
5. p.2-10: Again, γ should be defined.
6. p.2-14: might be better to add the symbol $\Rightarrow$ before the equation and a full stop after the equation.
7. p.2-41: might be better to add a full stop after the equation.
8. p.3-34: might be better to add a full stop after the equation.
9. p.4-2: the author uses $p(k, x)$ to denote the PDF, but in p.1 the notation used is $p_k(x)$.
10. p.4-6: might be better to add a full stop after the equation.
11. p.4-12: might be better to add a full stop after the equation.
12. p.4-13: Again, γ should be defined.
13. p.4-19: might be better to add a full stop after the equation.

14. p.4-29: might be better to add a full stop after the equation.

15. p.5-2: might be better to add a full stop after the equation.

16. p.5-5: might be better to add a full stop after the equation.

17. p.5-18: might be helpful to clarify that $\nu(k) > L_4(k)$ for all $k > 0$.

18. p.5-27: might be better to add a full stop after the equation.

19. p.5-31: might be better to add a full stop after the equation.

20. p.5-36: might be better to add a full stop after the equation.

21. p.6-8: might be better to add the symbol $\Rightarrow$ before the equation and a full stop after the equation.

22. p.6-11: might be better to add a full stop after the equation.

23. p.6-16: might be better to add the symbol $\Rightarrow$ before the equation and a full stop after the equation.

24. p.6-21: might be better to add a full stop after the equation.

25. p.6-29: might be better to add a full stop after the equation.

26. p.7-3: might be better to add a full stop after the equation.

27. p.7-9: might be better to add the symbol $\Rightarrow$ before the equation and a full stop after the equation.

28. p.7-12: might be better to add a full stop after the equation.

29. p.7-17: might be better to add the symbol $\Rightarrow$ before the equation and a full stop after the equation.

30. p.7-22: might be better to add a full stop after the equation.

31. p.7-27: might be "when $k < 1$" (as in the theorem statement) instead of $forallpositivek$.

32. p.8-1: might be better to add a full stop after the equation.

33. p.8-9: might be better to add a full stop after the equation.

34. p.8-25: might be better to add a comma after the equation.
35. p.8-26: might be better to add a comma after the equation.
36. p.8-27: might be better to add a comma after the equation.
37. p.8-28: might be better to add a comma after the equation.
38. p.8-29: might be better to add a full stop after the equation.
39. p.8-36: might be better to add a comma after the equation.
40. p.9-1: might be better to add a comma after the equation.
41. p.9-2: might be better to add a comma after the equation.
42. p.9-3: might be better to add a full stop after the equation.
43. p.10-1: might be better to add a full stop after the equation.
44. p.10-35: might be better to add a full stop after the equation.
45. p.11-1: might be better to add the symbol $\Rightarrow$ before the equation and a full stop after the equation.
46. p.11-4: might be better to add the symbol $\Rightarrow$ before the equation and a full stop after the equation.
47. p.11-8: might be better to add a full stop after the equation.
48. p.11-16: might be better to add a full stop after the equation.
49. p.11-20: might be better to add the symbol $\Rightarrow$ before the equation and a full stop after the equation.
50. p.11-22: avoid acronym like RHS and LHS without say its respective meaning.
51. p.11-27: might be better to add a full stop after the equation.
52. p.12-3: might be better to add a comma after the equation.
53. p.12-4: might be better to add a comma after the equation.
54. p.12-5: might be better to add a full stop after the equation.

55. p.12-10: might be better to add a full stop after the equation.

56. p.12-25: might be better to add a full stop after the equation.

57. p.12-38: might be better to add a full stop after the equation.

58. p.13-10: might be better to add a full stop after the equation.

59. p.13-15: might be better to add a full stop after the equation.

60. p.13-18 might be better to add a comma after the equation.

61. p.13-19: might be better to add a comma after the equation.

62. p.13-31: might be better to add a full stop after the equation.

63. p.14-19: might be better to add a full stop after the equation.

64. p.14-29: might be better to add a full stop after the equation.

65. p.15-3: might be better to add a full stop after the equation.

66. p.15-41: might be better to add a full stop after the equation.

4 References

1. BERG, C. and PEDERSEN, H. L. (2006). The Chen-Rubin conjecture in a continuous setting. *Methods and Applications of Analysis*, vol. 13, n. 1, p. 63-68.

2. BERG, C. and PEDERSEN, H. L. (2008). Convexity of the median in gamma distribution. *Arkiv för Matematik*, vol. 46, n. 1, p. 120-121.

3. CHEN, J. and RUBIN, H. (1986). Bounds for the difference between median and mean of gamma and Poisson distributions. *Statistics & Probability Letters*, vol. 4, n. 6, p. 281-283.

4. CHOI, K. P. (1994). On the medians of gamma distributions and an equation of Ramanujan. *Proceedings of the American Mathematical Society*, vol. 121, n. 1, p. 245-251.

5. GAUNT, R. E. and MERKLE, M. (2021). On bounds for the mode and median of the generalized hyperbolic and related distributions. *Journal of Mathematical Analysis and Applications*, vol. 493, n. 1, e124508.

6. LYON, R. F. (2021). On closed-form tight bounds and approximations for the median of a gamma distribution. *PLOS One*, vol. 16, n. 5, e0251626.

7. WILSON, E. B. and HILFERTY, M. M. (1931). The distribution of chi-square. *Proceedings of the National Academy of Sciences*, vol. 13, n. 1, p. 63-88.
