# Supplementary material for: Tight bounds for the median of a gamma distribution
Source: PLoS One. 2023 Sep 8;18(9):e0288601. doi: 10.1371/journal.pone.0288601 (PMC10490949; doi:10.1371/journal.pone.0288601)
Supplement: Attachment — Submitted filename: Rebuttal Letter.pdf [file pone.0288601.s003.pdf]

Rebuttal Letter / Response to Reviewers
by Richard F. Lyon
PONE-D-22-34782
24 June 2023

I appreciate the positive reviews from both reviewers.

I mostly made changes in line with their suggestions, and added a few more bits along the way to help clarify (such as the new section "Toward a proof" that might help others complete the work, and an example of a third-order rational function interpolation with low relative error).

Reviewer #1:

…The weaknesses I see are that the review of the literature is quite lacking (there are many works on very close topics such as asymptotic bounds for the gamma, Poisson and negative binomial distributions that are not mentioned, even very recent ones. Including them could help motivate the results of the paper a bit more and help the reader put the results in perspective.

I have added three references to very recent related works. But I don't understand them well enough to say much about how related they are, so I just said, "In addition to the works mentioned above, there have been several more recent works on asymptotic properties and bounds for medians and other quantiles of gamma distributions and of the closely related Poisson and negative binomial (or Polya or Pascal) distributions, with a variety of interesting approaches \cite{priore2022approximate, ouimet2023refined, pinelis2021monotonicity}.

… Also, the figures could benefit from a legend or a clearer labelling of the different curves.

I found that Figure 2 had an extra curve (from a corollary) that should not have been there, and that confused the interpretation of some of the labels on the curves, so I simply removed that (it was a thin solid red curve in the original figure). Now Figure 2 seems more clear.

For Figures 1 and 3 the labels on the curves seem clear and unambiguous.

That leaves Figure 4, which I agree was a bit confusing and crowded, with the (conjectured) interpolated bounds being described in the caption but not in the figure itself. So I managed to squeeze in a legend, and removed the 53% and 54% quantile curves that were in that area, leaving the presentation more symmetric between 48% and 52% and leaving more room for the legend.

Reviewer #2

…the paper needs minor revisions … my comments and suggestions below …

Most of the suggestions were of the form "*might be better to add a full stop after the equation*" or "*might be better to add a comma after the equation*". I followed all of those, but I think I also added another comma or two. In addition, many were "*might be better to add the symbol ⇒ before the equation and a full stop after the equation*", which I also agreed with and did. In each case I followed the typical LaTeX styling advice of separating the end punctuation from the math by a thin-space. Where I already had full stops after a few equations, I moved them away by a thin-space. Most of these changes are hard to spot in the diff, as the full stop or comma are too short for underlining to show up, and the blue color is easy to miss. But they're there.

I enumerate responses to the numbered suggestions (in *italics* below) that were more than these, and I use **bold** where I did something a bit contrary to reviewer #2's suggestion.

These 3 I group and treat out of order:

1. p.1-9: γ should be defined.
5. p.2-10: Again, γ should be defined.
12. p.4-13: Again, γ should be defined.

In the abstract I added "(with γ being the Euler--Mascheroni constant)". In the text, on p.2, I added "(with $\gamma \approx 0.5772157$ being the Euler--Mascheroni constant)" which includes its approximate value. Thank you for noticing this was needed. **I think it does not need to be said again on p.4.**

2. p.1-17: might be "...probability density function (PDF)..." instead of "...PDF...".

I took the liberty to add a possessive, this way: "The gamma distribution's probability density function (PDF) is …"

3. p.1-19: might be x>0 instead of x≥0. Note that if k=1 and x=0, we have p1(0) = 0/0, which is an indeterminate form.

Yes, x>0. Done.

4. p.1-23: might be "... cumulative distribution function (CDF)..." instead of "...CDF... ".

Done

9. p.4-2: the author uses p(k,x) to denote the PDF, but in p.1 the notation used is pk(x).

I switched to using the subscript k consistently for both the PDF p_k(x) and the CDF P_k(x).

17. p.5-18: might be helpful to clarify that ν(k) > L4(k) for all k > 0.

I added "for all k > 0" and also added some clarifying words to the start of the paragraph, "Since the denominator and the exponent are positive…". And I removed the redundant comment that followed: "The derivation holds for all k > 0."

31. p.7-27: might be "when k < 1" (as in the theorem statement) instead of for all positive k.

I fixed it the other way. The theorem statement now says for all positive k, as in the table of theorems to prove. I re-checked that the proof is valid over that full domain.

50. p.11-22: avoid acronym like RHS and LHS without say its respective meaning.

Reworded as "Dividing by the positive right-hand side gives the equivalent condition…".

Other changes I made, which you may notice in the diffs:

I changed "The positivity constraint is met" to "The constraint is met" in one place where the constraint inequality is not a comparison to 0.

Where I had "rather a" I changed to "rather than a".

I changed "which is completes the proof" to "this completes the proof" in the proof of Lemma 4.

In the Fig. 4 caption I used a code font (\texttt) for the Matlab function name gammaincinv.

I think that's all. I tried to restrain my tendency to keep tweaking.
